# Attitudes, Knowledge, and Worry About HIV in the U=U Era: A Campaign with Before-After Surveys Among HIV-Negative Men Who Have Sex with Men in Sweden

**DOI:** 10.1007/s10461-025-04972-9

**Published:** 2026-02-19

**Authors:** Carl Fredrik Sjöland, Nicklas Dennermalm, Lena Nilsson Schönnesson, Karin Laine, Erica Kanon, Daniel Suarez, Anna Mia Ekström

**Affiliations:** 1 Global and Sexual Health Research Group (GloSH), Department of Global Public Health, Karolinska Institutet, Stockholm, Sweden; 2 Public Health Agency of Sweden, Solna, Sweden; 3 Department of Social Work, Stockholm University, Stockholm, Sweden; 4 Posithiva Gruppen, Stockholm, Sweden; 5 Noaks Ark Stockholm, Stockholm, Sweden; 6 Department of Clinical Research and Education, South General Hospital, Stockholm, Sweden; 7 Department of Infectious Diseases/Venhälsan, South General Hospital, Stockholm, Sweden

**Keywords:** U=U, HIV stigma, Negative attitudes, HIV knowledge, HIV worry, Men who have sex with men, Sweden

## Abstract

Despite evidence that undetectable equals untransmittable (U=U), negative attitudes toward people living with HIV persist among men who have sex with men (MSM). We examined HIV-related knowledge, attitudes and relationship willingness among Swedish MSM and evaluated an anti-stigma campaign. Repeated cross-sectional online surveys of MSM aged ≥ 18 years were conducted in Sweden (2020–2021) before and after an anti-stigma campaign. Participants were recruited via LGBTQIA+ media and dating or cruising platforms. Multivariable logistic regression adjusted for sociodemographic and behavioral factors. We analyzed 3100 pooled responses and 1132 post-campaign responses. Knowledge gaps were substantial: 72% had not encountered U=U messaging and only 53% knew that treatment prevents transmission. Nearly half expressed unwillingness to consider relationships with people living with HIV. Negative attitudes decreased with higher knowledge (adjusted odds ratio [aOR] 0.38; 95% confidence interval [CI] 0.28–0.53) and increased with greater worry (aOR 3.66; CI 2.75–4.90). Greater relationship willingness was associated with higher HIV knowledge (aOR 3.79; CI 2.67–5.41). Less negative attitudes and greater relationship willingness were also linked to openness about sexual orientation. Campaign exposure was significantly associated with higher knowledge, less negative attitudes, lower worry, and greater relationship willingness. U=U awareness remains limited and stigma persists. The interrelations between knowledge, worry, and attitudes highlight cognitive and emotional dimensions of stigma. Observed links with campaign exposure suggest potential value of concise, context-specific U=U-centered communication in addressing stigma among MSM.

**Supplementary Information:**

The online version contains supplementary material available at 10.1007/s10461-025-04972-9.

## Introduction

Men who have sex with men (MSM) have been disproportionately affected by HIV since the epidemic’s onset, facing higher disease burden, and through association with HIV, increased stigma directed towards the full LGBTQIA+ community (lesbian, gay, bisexual, transgender, queer, intersex and expanding sexual identities). HIV-related stigma and discrimination persist even in high-income, LGBTQIA+ -friendly settings like Sweden, where antiretroviral treatment is readily available [1, 2].

UNAIDS defines HIV-related stigma as negative beliefs, feelings, and attitudes toward people living with HIV, while discrimination is unfair treatment based on one’s actual or perceived HIV status [3]. Such perceptions hinder effective prevention and care efforts; adversely affecting the mental well-being of people living with HIV; contributing to depression, social isolation, and reduced quality of life; with anticipation of stigma acting as a deterrent to HIV-testing [4, 5].

Despite ground-breaking treatment advancements and evidence that individuals with undetectable viral loads cannot transmit HIV—captured in the “Undetectable equals Untransmittable” (U=U) paradigm [6]—this knowledge has not eliminated stigma. In Sweden, over 95% of people living with HIV have undetectable viral loads, with MSM making up roughly a third of people living with HIV in Sweden [7]. National policies have promoted “treatment as prevention” since 2017 and fully endorsed U=U [8]. However, a recent survey revealed that 86% of the Swedish public are unaware that there is no risk of HIV transmission under U=U conditions [9].

Research often addresses manifestation of HIV-related stigma, including: structural and institutional stigma (particularly in healthcare settings); enacted stigma from the public [9, 10]; and the experiences of people living with HIV [1, 10]. Interventions typically target individuals at risk of or living with HIV to promote healthy behaviors. Research on what underlies stigma or which seeks to promote non-stigmatizing attitudes among HIV-negative populations is limited [11, 12]. A recent review highlights the lack of stigma-reduction studies outside clinical settings, also in high-income contexts and among HIV-negative key populations [1]. Further, the interplay of emotion and cognition to shape negative HIV-related attitudes remains understudied outside specific venues of stigma. Many frameworks emphasize the downstream impacts of stigma, tending to overlook upstream drivers [1]. The Health Stigma and Discrimination Framework (HSDF) is a notable exception, providing a comprehensive model that highlights both the drivers and manifestations of stigma [13].

## Conceptual Framework

Our conceptual framework, adapted from a dual-process model [14] and the HSDF [13], positions negative attitudes as influenced by both cognitive (knowledge) and affective (worry) processes, and manifested behaviorally through willingness to form or enter a relationship with a partner living with HIV (relationship willingness). The model assumes bidirectional relationships among these domains: knowledge may reduce worry and negative attitudes; worry may amplify stigma, avoidance, and relationship willingness reflects both cognitive understanding and emotional acceptance.

**Fig. 1:**
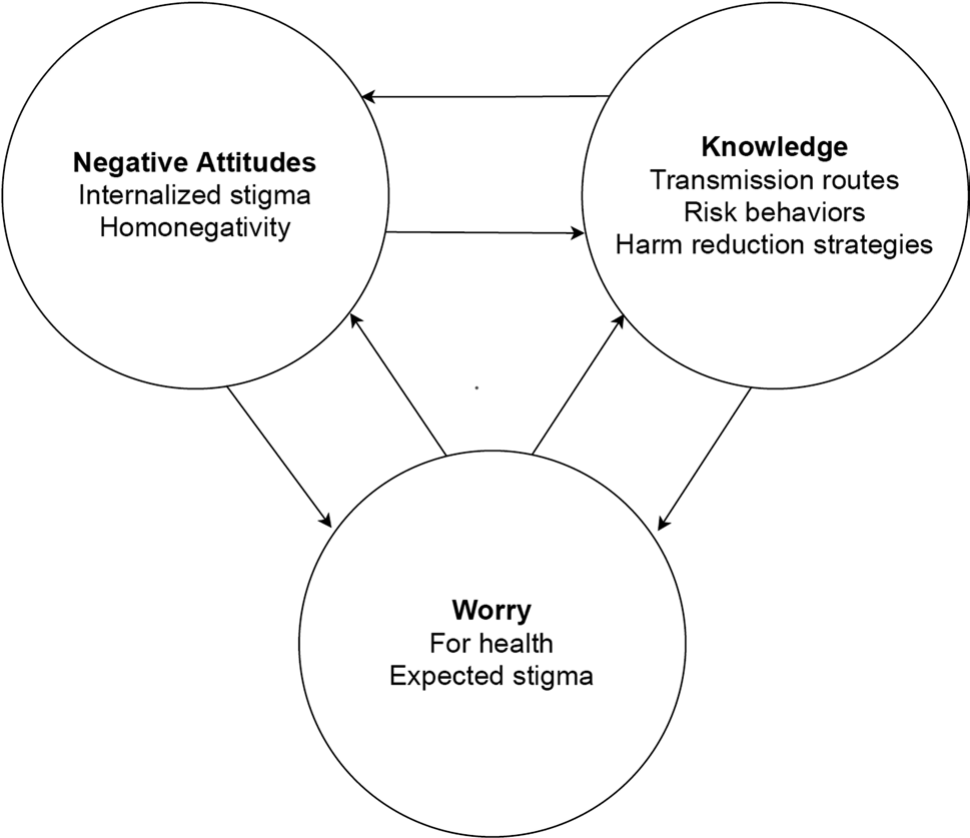
Conceptual framework illustrating the bidirectional relationships between HIV-related negative attitudes, HIV knowledge, and HIV-related worry or transmission concerns

The conceptual framework (Fig. 1), posits bidirectional relationships among three key domains:

- HIV-related Negative Attitudes: unfavorable beliefs or emotional responses toward people living with HIV.
- HIV Knowledge: accurate understanding of HIV transmission, prevention, and the U=U principle.
- HIV-related Worry and Transmission Concerns: Anxiety or concern about acquiring or transmitting HIV.

Relationship willingness is treated as a behavioral manifestation of these domains, reflecting how cognitive and affective processes translate into interpersonal attitudes and decisions.

We hypothesize that these domains influence one another bidirectionally. For instance, increased worry about HIV may motivate information seeking and enhance knowledge; greater knowledge, particularly of U=U, may reduce negative attitudes; negative attitudes may amplify worry and avoidance, undermining relationship willingness.

### Study Aims

We aim to assess the prevalence of negative attitudes toward HIV and people living with HIV among HIV-negative MSM in Sweden and to identify associated cognitive, emotional, and behavioral factors. Specifically, we examine the roles of HIV-related knowledge, worry concerning transmission, and willingness to form relationships with people living with HIV. We also evaluate the impact of an anti-stigma campaign that draws parallels between homophobia and HIV-related stigma.

By exploring these interconnections among HIV-negative MSM in Sweden, we aim to clarify how cognitive, emotional, and behavioral dimensions interact with societal influences to sustain stigma. The study aims to complement and expand upon those from our simultaneous qualitative study [15].

## Methods

We conducted a repeated cross-sectional, Internet-based survey among Swedish MSM during two periods: June–August 2020 and March–June 2021. Inclusion criteria were self-identification as MSM, being at least 18 years old, and residing in Sweden.

Participants were recruited through convenience sampling. The survey was disseminated through multiple channels frequented by MSM, including an LGBTQIA+ news site: *QX.se*; MSM dating or cruising geo-social networking platforms: *Qruiser.com* and *Grindr*; and targeted posts on the Instagram and Facebook pages of HIV organizations. Potential participants were directed to the website *hivattityd.se,* which was active for the duration of the study. The *Qualtrics* survey platform was used, where built-in quality control functions were applied to prevent duplicate responses [16]. The study was approved by the Swedish Ethical Review Authority (dnr 2020–00975). Before initiating the survey, participants received detailed information about the study’s purpose, procedures, and data protection measures. Informed consent was obtained electronically prior to voluntary and anonymous participation.

**Fig. 2:**
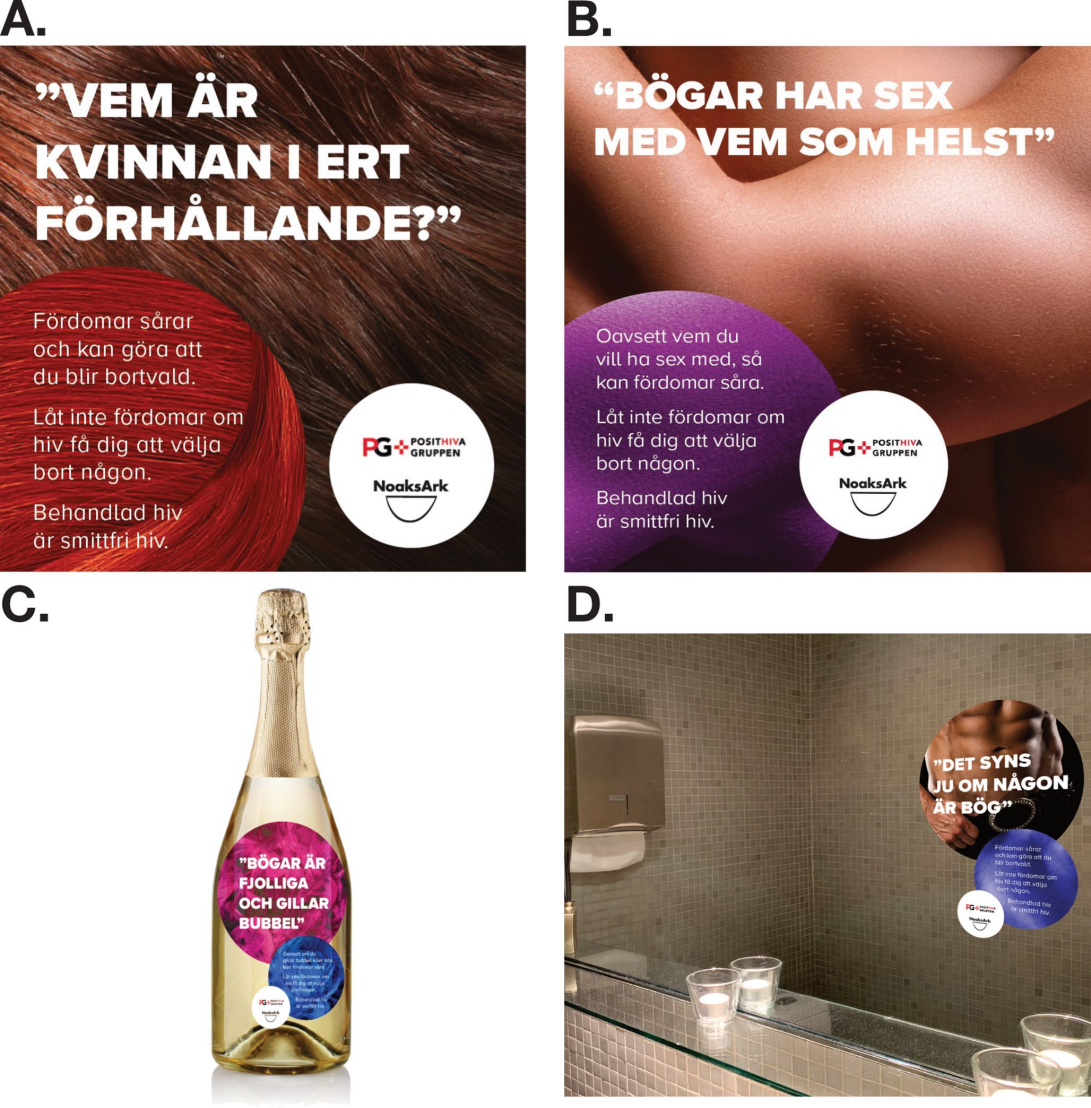
Materials from the anti-stigma campaign, including translations. The messaging aims to poke fun at stereotypically uninformed views about MSM, sarcastically tying together well-known tropes toward the gay community with prejudice about people living with HIV. Each installment includes a message urging not to reject people due to HIV status and where to access information about U=U. The term ‘smittfri hiv’ is used throughout. A. "Who is the woman in your relationship? - Prejudice hurts and may cause you to be rejected. Do not let prejudice about HIV make you reject someone. Treated HIV is non-infectious HIV." B. "Gays have sex with anyone - No matter who you want to have sex with, prejudice can hurt. Do not let prejudice about HIV make you reject someone. Treated HIV is non-infectious. C. "Gays are sissies and like sparkling wine" "You can see if someone is gay - Whether or not you like sparkling wine, prejudice can hurt. Do not let prejudice about HIV make you reject someone. Treated HIV is non-infectious HIV." D. "You can see if someone is gay - Prejudice hurts and may cause you to be rejected. Do not let prejudice about HIV make you reject someone. Treated HIV is non-infectious HIV."

**Fig. 3:**
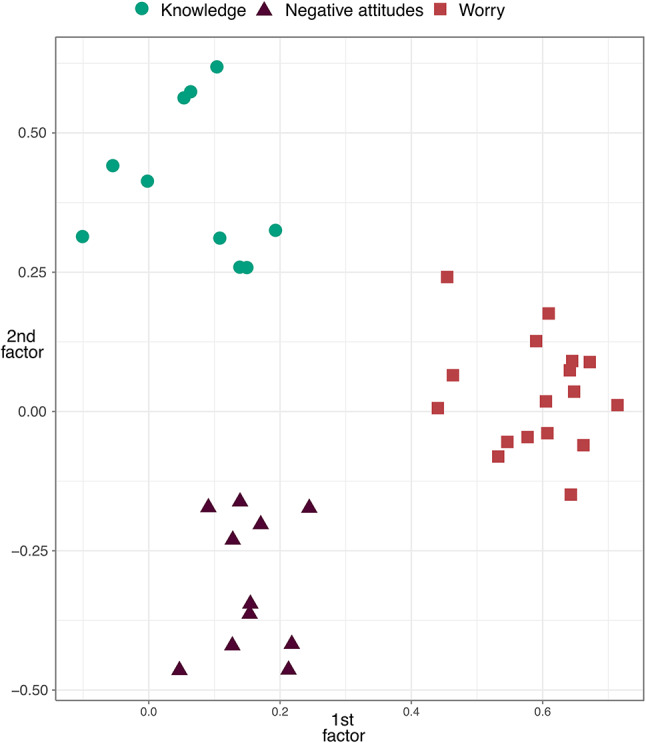
Index validation: Loading plot of principal axis factoring (PAF) components. Loading plot showing eigenvectors of the first and second factors from the PAF using 2021 questions from all three indices. Each point represents an item loading, colored by predefined domain (negative attitudes, HIV knowledge, and HIV-related worry). Items grouped spontaneously within their domains with minimal overlap, consistent with results of k-means clustering (average silhouette 0.76; Calinski–Harabasz 221; adjusted Rand index 1.00), confirming the structural validity of the three indices.

An anti-stigma campaign initiated in January 2021, between the two survey rounds, and ran throughout the second survey period. It was disseminated in LGBTQIA+ online spaces and at select locations frequented by MSM, primarily LGBTQIA+ nightlife venues around Stockholm, with materials remaining publicly available for an extended period thereafter. The campaign was intentionally provocative, linking negative attitudes toward people who are living with HIV with well‑known homophobic tropes (Fig. 2). The campaign aimed to raise awareness of HIV‑related stigma and to reduce negative attitudes toward people who are living with HIV among HIV-negative MSM. Its design drew on insights from the first round of this study as well as from our concurrent qualitative study [15]. Accordingly, the campaign used the term “*smittfri hiv*” (non‑infectious HIV) as the most recognizable and accepted Swedish translation of U = U messaging. Because the survey was anonymous, responses could not be linked across rounds; in round 2, participants were asked whether they had previously responded to round 1. No formal sample‑size calculation was undertaken; the goal was to recruit as many participants as possible (Fig. 3). 

### Survey Instrument

The survey was co-developed by our research team, comprising HIV clinicians, researchers, and representatives from HIV organizations, including MSM living with HIV. Iterative refinement to ensure clarity and relevance followed from two rounds of online video-call pilot tests with anonymous MSM recruited by the partner organizations. The survey, available in Swedish, English, and Spanish, had an estimated completion time of 15–20 min.

Survey modules included: demographics; sexual orientation and practices; and attitudes, knowledge, and worry related to: HIV, becoming HIV-positive, and about people living with HIV. Minor refinements were made to the module on HIV-related worry for the second round based on our qualitative findings.

### Measures

Negative HIV-related attitudes were assessed through survey items measuring unfavorable beliefs and feelings toward people living with HIV, such as agreeing with stigmatizing statements.

HIV Knowledge was evaluated based on correct responses to questions about HIV transmission, prevention methods (including pre-exposure prophylaxis [PrEP] and post-exposure prophylaxis [PEP]), and understanding of U=U.

HIV-related worry was measured through questions on concerns of acquiring HIV (medical and social effects) and concerns about further transmission. (See Online Appendix 1 for detailed index question coding).

Willingness to form a relationship with someone living with HIV was dichotomized from responses to the question “Do you think you could have a relationship with someone living with HIV?”, dichotomized as either “yes” or “no/doubtful/no response”.

Covariates included age (categorized in 10-year brackets), country of birth (Sweden or other), education level (university: yes/no), relationship status (steady partner, open relationship, no partner), use of dating or cruising apps (yes/no), number of sexual partners in the past year (0, 1–2, 3–5, 6 +), and city size (Stockholm, other major city [Gothenburg, Malmö, Uppsala], medium-sized city [50,000–200,000 inhabitants], smaller city [10,000–49,999 inhabitants], small town/countryside [< 10,000 inhabitants]).

Sexual identity and openness were combined into a composite variable reflecting both self-identified sexual orientation and degree of disclosure. Sexual identity was based on responses to “How do you identify?” (homosexual, bisexual/pansexual, or other), and openness was dichotomized from a question on openness about one’s sexuality to others, with response options: (1) always hide it, (2) hide it most of the time, (3) mostly open, and (4) always open. Responses 1–2 were categorized as not open and 3–4 as open. The resulting composite variable included six categories: homosexual and open, homosexual and not open, bisexual/pansexual and open, bisexual/pansexual and not open, other and open, and other and not open.

Additional covariates included acknowledgment that HIV stigma exists among MSM (yes/no), and ever having had sex with a partner living with HIV (yes/no).

### Data Analysis

Of 4,190 unique responses, 3,731 (89%) completed the full survey. Respondents who self-reported living with HIV (n = 334) were excluded. Because the survey was anonymous, respondents could not be linked across rounds. Those indicating prior participation were therefore excluded from pooled analyses to prevent duplication (n = 278), though retained in round-specific analyses. Analyses were restricted to cases with non-missing data on key exposures used in index construction or core covariates. No imputation was performed. This resulted in a pooled analytical sample of 3,100 unique respondents (2,244 from round 1 and 856 from round 2), and a round-specific sample of 1,132 for the 2021 campaign analyses.

Responses to survey items on HIV-related attitudes, knowledge, and worry were recoded into binary variables. One worry item was originally ordinal (Likert scale) and was dichotomized at the median for inclusion in indices. A complete list of survey items and the coding, including all variables used in index construction and as covariates, is provided in Online Appendix 1. These were summarized in composite indices using principal component analysis (PCA) for each domain, retaining the first component as indices for attitudes, knowledge, and worry after assessing variance explained by each component.

To ensure comparability due to changes between the first and second round’s questions on worry (adjusting to better align with our qualitative findings), round-specific PCAs were used for this index, which were standardized before being combined into an overall worry index. For analysis of factors associated with willingness to enter a relationship with someone living with HIV, a separate index for negative attitudes was created, by excluding this question from the attitude index PCA.

Construct validity of the indices was evaluated using confirmatory factor analysis, assessed using the Comparative Fit Index (CFI) and the Root Mean Square Error of Approximation (RMSEA). The index metrics were interpreted according to established standards and recommended cut-offs [17]. To further verify that items from the three index domains grouped as theoretically expected, we performed k-means clustering (k = 3) of two-factor principal axis factoring (PAF) loadings, which included all variables used in the three indices. Cluster separation was summarized by the average silhouette width and the Calinski–Harabasz index, and agreement with the predefined domains was quantified using the adjusted Rand index. This step served as a descriptive check of the agreement of our predefined domains with an unsupervised data-derived structure identified through PAF.

We conducted univariate descriptive analyses of relevant variables, followed by multivariable logistic regression using four pre-specified models corresponding to the main outcome domains: (1) negative HIV-related attitudes, (2) HIV knowledge, (3) HIV-related worry, and (4) willingness to form a relationship with a person living with HIV. Each model used the respective outcome, index or binary indicator, as the dependent variable and included the remaining indices and covariates described above, and which were selected based on the conceptual framework and prior literature. Indices for outcomes 1–3 were dichotomized at the median when used as dependent variables and divided into quintiles when used as independent variables to evaluate dose-dependent associations (i.e., progressive and monotonic change in the outcome with increasing exposure).

In second-round-only (2021) multivariable logistic regression models the campaign exposure variable was included. The primary aim was here to determine the association of campaign exposure and the index scores for attitudes, knowledge, and worry were therefore entered as z-standardized continuous variables to maximize power and comparability, whereas willingness to form a relationship was modeled as binary (yes and no/doubtful/no response).

Sensitivity analyses included per-round multivariable logistic regression models with identical covariates, as well as two models that excluded all respondents with “Do not want to answer” responses to any of the index questions or who responded in this manner on more than two items. Multicollinearity was evaluated using variance inflation factors (VIF). Statistical significance was assessed using 95% confidence intervals (CIs).

Statistical analyses were conducted using Stata 19.5 SE and R 4.4.5 using the *forestmodel* package 0.6.2 for visualization of regression models.

## Results

In total, 3,100 HIV-negative MSM participated in the study, with 60% having attended university and 24% reporting past sexual encounters with a partner living with HIV. Most respondents were homosexual and open about their sexual orientation (50%) and resided in urban areas (86%). The majority (86%) reported using dating or cruising apps. These findings align with expected demographic profiles and characteristics of the MSM-population in a high-income country like Sweden. Table 1 provides detailed demographics of the pooled analytic sample, with Online Appendix 2 detailing bivariate distributions across the four outcomes which were included in the pooled multivariable regression models (Table 2).

**Table 1:** Sociodemographic and behavioral characteristics of HIV-negative MSM included in the pooled analytic sample (N = 3100)

Characteristic	n (%)
*Knowledge index (quintiles)*	
Lowest	579 (19%)
Lower	649 (21%)
Middle	628 (20%)
Higher	619 (20%)
Highest	625 (20%)
*Worry index (quintiles)*	
Lowest	590 (19%)
Lower	638 (21%)
Middle	627 (20%)
Higher	623 (20%)
Highest	622 (20%)
*Negative attitude index (quintiles)*	
Lowest	893 (29%)
Lower	656 (21%)
Middle	518 (17%)
Higher	504 (16%)
Highest	529 (17%)
*Would consider relationship with someone living with HIV*	
No / Doubtful / No Response	1,487 (48%)
Yes	1,613 (52%)
Country of birth	
Sweden	2,623 (86%)
Other	427 (14%)
No response	50
*University education*	
No university	1,234 (40%)
Any university	1,844 (60%)
Unknown	22
*Relationship status*	
Steady partner	997 (32%)
Open relationship	620 (20%)
No partner	1,483 (48%)
*Use of dating or cruising apps*	
Non-user	434 (14%)
User	2,645 (86%)
No response	21
*Sexual partners (past year)*	
None	391 (13%)
1–2	1,153 (37%)
3–5	731 (24%)
6 +	825 (27%)
*Age group (years)*	
18–24	150 (5%)
25–34	418 (14%)
35–44	549 (19%)
45–54	635 (22%)
55–64	695 (24%)
65 +	459 (16%)
No response	194
*Urbanity*	
Stockholm	1,209 (39%)
Major city (Gothenburg, Malmö, Uppsala)	483 (16%)
Medium-sized city (50,000–200,000)	528 (17%)
Smaller city (10,000–49,999)	422 (14%)
Small town / Countryside (< 10,000)	457 (15%)
No response	1
Acknowledges HIV-stigma among MSM	1,927 (62%)
Ever had sex with an HIV-positive partner	735 (24%)
*Openness by sexual orientation*	
Homosexual & Open	1,562 (50%)
Homosexual & Not Open	337 (11%)
Bi/Pansexual & Open	222 (7%)
Bi/Pansexual & Not Open	837 (27%)
Other & Open	80 (3%)
Other & Not Open	62 (2%)

Percentages are calculated from the pooled analytic sample of HIV-negative MSM (N = 3100). Minor variation in denominators is due to item non-response. Detailed bivariate distributions across outcomes (negative attitudes, HIV knowledge, HIV-related worry, and relationship willingness) are presented in Online Appendix 2

### Prevalence Findings

A majority (72%) of respondents had not encountered any U=U messaging. Only 53% were aware that antiretroviral treatment reduces the risk of HIV transmission. Furthermore, only 41% knew that the duty to inform sexual partners about one’s positive HIV status could be waived when having an undetectable viral load, and just 25% were aware that the legal obligation to use a condom could be removed under the same conditions, provided for under the Swedish Communicable Disease Act and Communicable Diseases Ordinance. In the 2021 survey, just over half of respondents (610/1132) reported exposure to the anti-stigma campaign. (Online Appendix 3*for full responses to individual questions*).

### Construct Validity and Clustering of Index Domains

Construct validity tests indicated highly robust measures for the HIV-related worry indices, with Comparative Fit Index (CFI) values of 0.997 for 2021 and 0.980 for 2020, and Root Mean Square Error of Approximation (RMSEA) values of 0.014 for 2021 and 0.057 for 2020. The measures for HIV-related negative attitudes and HIV-related knowledge showed moderate construct validity, with CFI values of 0.771 and 0.850, and RMSEA values of 0.096 and 0.121, respectively. K-means clustering of the two-factor PAF loadings from 2021, as shown in Fig. 3, produced three clearly separated clusters (average silhouette = 0.76; Calinski–Harabasz = 221), which corresponded exactly to the predefined domains (adjusted Rand index = 1.00). In all PCA-derived indices, the first component (retained as the index) accounted for more than twice the variance explained by the second component.

### Multivariable Regression Results

Dose-dependent relationships in this section are expressed with comparisons between first and fifth quintiles, unless otherwise stated (full results in Table 2).

**Table 2:**
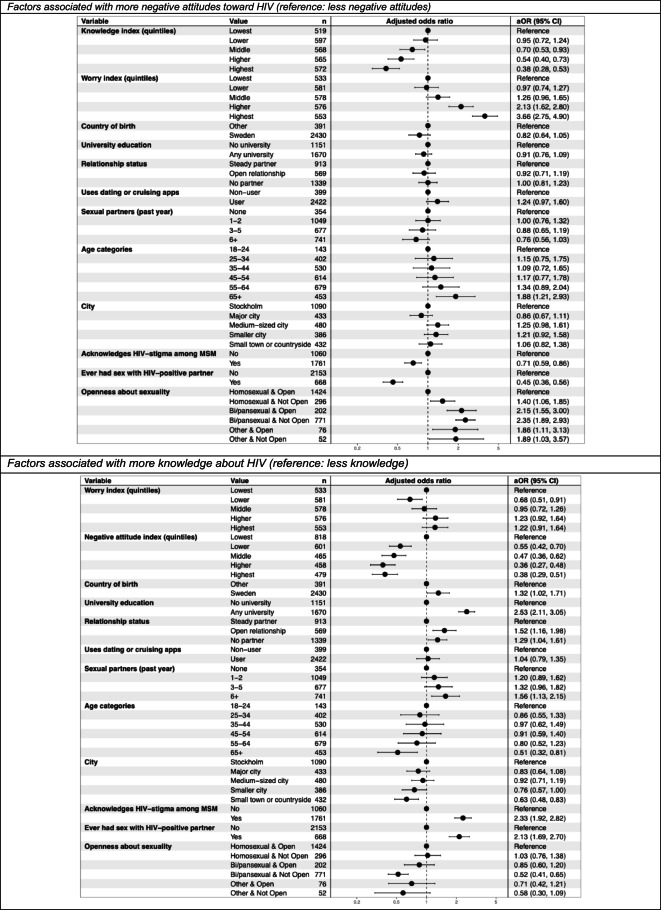

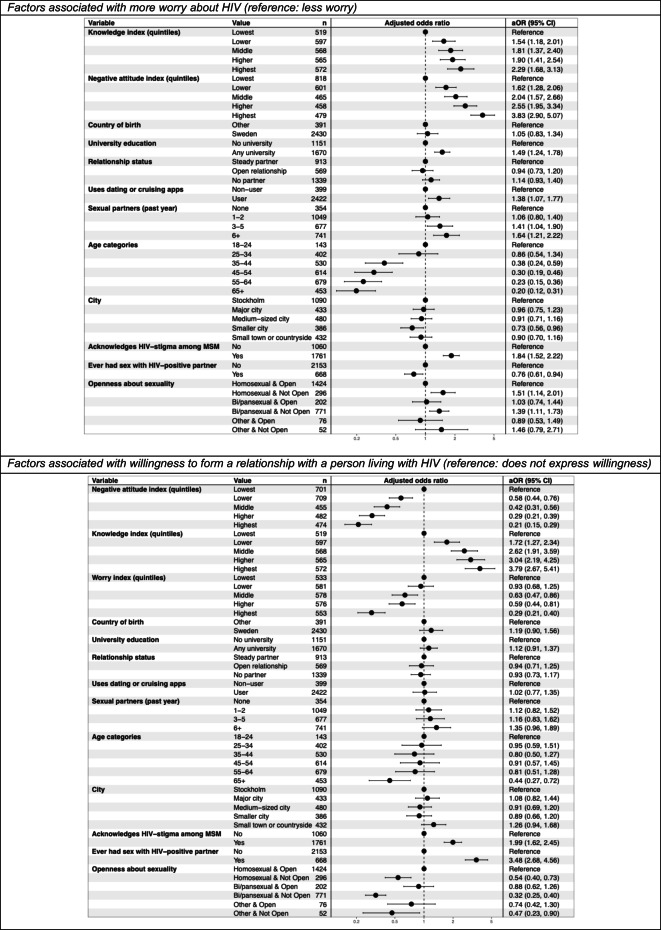
Multivariable logistic regression models showing adjusted odds ratios (aORs, 95% CIs) for factors associated with HIV-related attitudes, knowledge, worry, and relationship willingness among HIV-negative MSM in Sweden

When negative attitudes toward HIV or people living with HIV were the dependent variable, several factors were associated with more negative attitudes. There was a dose-dependent association with lower HIV knowledge (adjusted odds ratio [aOR] = 0.38; 95% confidence interval [CI]: 0.28–0.53) and higher HIV-related worry (aOR = 3.66; 95% CI: 2.75–4.90). Additionally, being over 65 years of age was associated with more negative attitudes (aOR = 1.88; 95% CI 1.21–2.93). Participants who acknowledged the presence of HIV stigma among MSM (aOR = 0.71; 95% CI 0.59–0.86) and those who had ever had sex with a partner living with HIV (aOR = 0.45; 95% CI 0.36–0.56) reported less negative attitudes. By contrast, compared with homosexual participants who were open about their sexuality, individuals identifying as homosexual and not open (aOR = 1.40, 95% CI 1.06–1.85); as bisexual or pansexual, who were open (aOR = 2.15, 95% CI 1.55–3.01) or not open (aOR = 2.35, 95% CI 1.89–2.93); and those of other sexual orientations, whether open (aOR = 1.86, 95% CI 1.12–3.13) or not open (aOR = 1.89, 95% CI 1.03–3.57); had more negative HIV-related attitudes.

Higher HIV knowledge was associated with several factors. There was a dose-dependent relationship with lower HIV-related negative attitudes (aOR = 0.38; 95% CI 0.29–0.51), though no consistent dose response was observed for worry, only those with lower worry had significantly less knowledge (aOR = 0.68; 95% CI 0.51–0.91). Knowledge was higher among participants born in Sweden (aOR = 1.32; 95% CI 1.02–1.71), those with a university education (aOR = 2.53; 95% CI: 2.11–3.05), and those in an open relationship (aOR = 1.52; 95% CI 1.16–1.98) or with no steady partner (aOR = 1.29; 95% CI 1.04–1.61), compared to those with a steady partner. There was also a dose-dependent relationship with the number of sexual partners (aOR = 1.56; 95% CI 1.13–2.15 comparing 0 with ≥ 6 partners). Conversely, participants aged 65 years or older had lower odds of knowledge (aOR = 0.51; 95% CI 0.32–0.81), as did those living in smaller towns or rural areas (aOR = 0.63; 95% CI: 0.48–0.83). Acknowledging HIV stigma among MSM (aOR = 2.33; 95% CI 1.92–2.82) and having ever had sex with a partner living with HIV (aOR = 2.13; 95% CI 1.69–2.70) were also associated with higher odds of knowledge. Participants identifying as bisexual or pansexual who are not open about their sexuality had lower odds of knowledge (aOR = 0.52; 95% CI 0.41–0.65) compared to open homosexual men.

When HIV-related worry was the dependent variable, higher worry was associated with both higher HIV knowledge (aOR = 2.29; 95% CI 1.68–3.13) and more negative HIV-related attitudes (aOR = 3.83; 95% CI: 2.90–5.07) in a dose-dependent manner. Odds of worry increased among those with university education (aOR = 1.49; 95% CI 1.24–1.78), using dating or cruising apps (aOR = 1.38; 95% CI 1.07–1.77), and number of sexual partners (aOR = 1.64; 95% CI 1.21–2.22 comparing 0 with ≥ 6 partners). Whereas worry decreased with age, showing an inverse dose-dependent response (aOR = 0.20; 95% CI 0.12–0.31 for ages 65 + vs. 18–24). Participants living in smaller cities also had lower odds of worry (aOR = 0.73; 95% CI 0.56–0.96). Acknowledging HIV stigma among MSM was associated with worry (aOR = 1.84; 95% CI 1.52–2.22). Compared with homosexual men who are open about their sexuality, being homosexual and not open (aOR = 1.51; 95% CI 1.14–2.01) or bisexual/pansexual and not open (aOR = 1.39; 95% CI: 1.11–1.73) was associated with higher worry.

Regarding the willingness to enter a relationship with someone living with HIV, there was a strong dose-dependent association with both lower HIV-related negative attitudes (aOR = 0.21; 95% CI 0.15–0.29) and higher HIV knowledge (aOR = 3.79; 95% CI 2.67–5.41). Higher worry was inversely associated with willingness (aOR = 0.29; 95% CI 0.21–0.40). Participants aged 65 years or older had lower odds of willingness (aOR = 0.44; 95% CI 0.27–0.72), while those who acknowledged HIV stigma among MSM (aOR = 1.99; 95% CI 1.62–2.45) and those who have ever had sex with a partner living with HIV (aOR = 3.48; 95% CI 2.68–4.56) had higher odds of willingness. Compared with open homosexual men, all participants who were not open showed lower odds of willingness, including homosexual and not open (aOR = 0.54; 95% CI 0.40–0.73), bisexual/pansexual and not open (aOR = 0.32; 95% CI 0.25–0.40), and other and not open (aOR = 0.47; 95% CI 0.23–0.90).

### Impact of the Anti-stigma Campaign

In analyses restricted to 2021 data, adjusting for sociodemographic and behavioral covariates, exposure to the anti-stigma campaign was associated with lower negative attitudes (aOR = 0.69; 95% CI 0.50–0.95), higher HIV knowledge (aOR = 1.53; 95% CI 1.10–2.13), lower HIV-related worry (aOR = 0.74; 95% CI 0.55–0.98), and greater willingness to form a relationship with a person living with HIV (aOR = 1.56; 95% CI 1.09–2.24), compared with participants who had not seen the campaign (Table 3; Online Appendix 4 for full model results).

**Table 3:** Separate multivariable logistic regression on attitudes, knowledge, worry related to HIV and ready to consider relationship with person living with HIV among HIV-negative MSM of whom 54% (592/1099) had seen the anti-HIV stigma campaign targeting MSM in Stockholm 2021

Outcome	Seen campaign	Adjusted odds ratio (95% CI)
Negative attitudes	Yes vs. No	0.69 (0.50–0.95)
Knowledge	Yes vs. No	1.53 (1.10–2.13)
Worry	Yes vs. No	0.74 (0.55–0.98)
Relationship willingness	Yes vs. No	1.56 (1.09–2.24)

Each model was adjusted for similar sociodemographic and behavioral covariates as in pooled models. Full regression outputs for all exposures and covariates are presented in Online Appendix 4.

### Sensitivity Analyses

Sensitivity analyses using round-specific multivariable logistic regression models yielded associations consistent in both magnitude and direction when compared to those from the final models. Similarly, excluding respondents with “Do not want to answer” responses on items used to construct the indices (more than two items [n = 2,978] or on any item [n = 2,340]) produced similar estimates in direction and relative magnitude. Confidence intervals in both sets of sensitivity analyses were wider due to lower statistical power, which affected marginally significant estimates but did not alter the overall interpretation of findings. Variance inflation factor (VIF) values indicated minimal multicollinearity across all models, including all sensitivity analyses.

## Discussion

Our study finds robust associations between HIV-related negative attitudes, knowledge, and worry. In adjusted models, higher knowledge about HIV and U=U is associated with less negative attitudes and greater willingness to consider relationships with people who are living with HIV, both with clear dose-dependent gradients. At the same time, worry operates as a seemingly independent pathway: higher worry is dose-dependently associated with more negative attitudes and lower relationship willingness. These patterns support the dual‑process framework in which cognitive and affective processes can move in different directions [14].

The findings further suggest that among HIV-negative MSM, attitudes toward people living with HIV can frequently be shaped by unfounded worries, which persist and shape perception independently of knowledge or awareness of U=U. Despite national endorsement of treatment as prevention in Sweden, U=U awareness remains incomplete in this community [6]. This reinforces how affective responses, such as worry or fear of infection, could act to maintain stigma even when cognitive understanding of transmission risks is accurate. This aligns with our qualitative research in Sweden, which found that societal influences, internalized stigma, and moral framings surrounding responsibility and risk contribute to persistent fears and skepticism toward U=U, even among those who acknowledge the scientific validity [15]. Earlier studies have reported similar links between emotion, misinformation, and stigmatizing attitudes [18, 19], yet few have quantified them in key populations. By demonstrating that HIV-related worry is linked to attitudes and not only knowledge, our study clarifies potential mechanisms through which stigma endures in the U=U era and illustrates how emotional and cognitive processes can interact to sustain negative perceptions of people living with HIV.

Higher HIV knowledge is associated with less HIV-related negative attitudes, underscoring the role of education and U=U communication in reducing stigma [12]. Yet, increased HIV-related worry remains independently associated with more negative attitudes, even after adjusting for knowledge, indicating that emotional and cognitive pathways appear to operate in parallel. Higher worry was also associated with a greater number of sexual partners in the past year, suggesting that worry may be influenced not only by misinformation, but also by perceived behavioral risk. This may reflect a feedback loop in which sexual activity heightens concern about HIV acquisition, prompting both information-seeking and anxiety, particularly among younger MSM.

Substantial proportions of respondents also reported persistent worry: 41% expressed concern about HIV transmission and 24% about death, indicating a potential gap between knowledge and emotional acceptance of medical advances such as U=U. These findings align with prior work showing that information deficits alone cannot explain stigma, as anxiety and mistrust toward biomedical prevention continue to shape attitudes [20–22]. Worries persisted even among those with higher education, suggesting that education does not translate into reassurance. Anti-stigma strategies should therefore target both rational understanding and affective fears, addressing underlying insecurities and moral framings around responsibility and transmission—an interpretation also supported by our qualitative research in Sweden [15].

Despite targeted interventions, substantial knowledge gaps also remain among MSM regarding PrEP, PEP, and U=U. Although HIV knowledge within the MSM community remains higher than in the general Swedish population [9, 22, 23], there is a need for sustained, comprehensive education. This is particularly evident in the limited understanding of evidence-based provisions such as the potential waiving of condom-use obligations and status-disclosure mandates for people living with HIV who maintain long-term undetectable viral loads [8]. These policies are intended to balance biomedical risk reduction with stigma prevention, a goal weakened by limited public understanding of their rationale.

Consistent with prior research, our findings show that greater HIV knowledge—especially understanding of U=U—is strongly associated with increased willingness to engage in romantic or sexual relationships with people living with HIV, whereas higher HIV-related worry correlates with lower willingness [6]. Despite sustained U=U promotion in Sweden, misconceptions remain common, including the belief that an undetectable viral load merely indicates “low” rather than “no” transmission risk, reinforcing uncertainty and mistrust toward treatment effectiveness [4, 24]. Such skepticism perpetuates a “disclosure double standard,” whereby individuals living with HIV risk rejection when disclosing their status, despite posing no transmission risk under U=U conditions [20]. These patterns mirror the dynamics of stigma associated with disclosing one’s sexual orientation, illustrating how incomplete knowledge and social prejudice intersect to sustain barriers to acceptance of both people living with HIV and LGBTQIA+ identities.

Openness about one’s sexual orientation, whether identifying as gay or bisexual, was consistently associated with fewer HIV-related negative attitudes, greater HIV knowledge, and increased willingness to engage in relationships with people living with HIV, without a corresponding rise in HIV-related worry. These findings suggest that outness, as an indicator of self-acceptance and social integration, may play a protective role against expressing stigmatizing attitudes. Conversely, participants who were not open about their sexual orientation, particularly bisexual and pansexual men, exhibited more negative attitudes, lower knowledge, and greater worry.

This pattern underscores the psychological and social dimensions linking HIV stigma with broader societal attitudes toward homosexuality and MSM. Prior studies have described how internalized homonegativity and anticipated stigma can shape perceptions of both HIV and sexual identity [21, 25]. In this context, fear of disclosing either HIV status or sexual orientation may overlap and lead some individuals to adopt stigmatizing attitudes as a form of self-distancing or social protection. Addressing these intertwined stigmas requires interventions that not only communicate biomedical facts, but also foster acceptance and visibility within LGBTQIA+ communities.

In line with previous work, the variability in global U=U comprehension and in what constitutes acceptable and effective communication underscores the need for context-specific, memorable messaging [26]. In our study, exposure to the anti-stigma campaign—linking homophobia and HIV prejudice—was associated with more positive attitudes, higher HIV knowledge, greater willingness to form relationships with people living with HIV, and less HIV-related worry. While causality cannot be inferred from these cross-sectional data, this pattern suggests that well-targeted campaigns can effectively challenge stigma, prompt reflection, and alleviate concern surrounding HIV transmission. Such resonant, contextually-grounded approaches may therefore complement factual U=U communication in advancing stigma reduction among MSM in Sweden.

Finally, we find support for our conceptual model of distinct yet interrelated cognitive and affective domains underlying HIV stigma. The psychometric analyses confirmed construct validity for the indices, and the unsupervised clustering of item loadings showed that questions within each domain grouped spontaneously and with clear separation. This coherence strengthens our confidence that the indices reflect distinct constructs, suggesting that the conceptual framework and its measures capture stable underlying dimensions.

### Strengths and Limitations

To our knowledge, this study is among the first large-scale quantitative investigations of factors associated with HIV-related negative attitudes, knowledge, and worry among HIV-negative MSM. Sensitivity analyses demonstrated consistent results between survey rounds, supporting the reliability and robustness of the findings.

Although convenience sampling and the online, anonymous format may limit generalizability to all MSM, the large and heterogeneous sample strengthens external validity. Anonymity and ease of access likely facilitated participation from otherwise hard-to-reach subgroups and encouraged candid responses. High reported use of dating or cruising apps likely reflects this online recruitment context rather than overestimation of prevalence among all MSM. Participant demographics were consistent with expectations for MSM in high-income settings, with overrepresentation of highly educated and urban individuals, but also included substantial participation from older age groups and those in steady or monogamous relationships—suggesting broad coverage of perspectives within the MSM community.

The surveys were conducted during the COVID-19 pandemic, which may have influenced response behavior and campaign exposure. In Sweden, minimal restrictions and voluntary isolation likely increased online engagement, potentially enhancing recruitment. However, the anonymous and cross-sectional design precluded individual follow-up and causal attribution of observed attitude shifts to the campaign. Survey fatigue was likely limited in Sweden, which had fewer large-scale public surveys during this period, and several respondents noted that they appreciated a survey unrelated to COVID-19.

The campaign itself may have appealed more to individuals already holding less negative attitudes, introducing potential selection bias. Nonetheless, the substantial reporting of stigmatizing views suggests that social desirability bias was limited. Minor acquiescence bias may have arisen from the phrasing of some knowledge and worry items, which could restrict cross-population comparability, although any resulting misclassification is likely non-differential within the study.

Construct validity testing showed very high internal consistency for the worry index and acceptable fit for the attitude and knowledge indices, indicating scope for further refinement of the latter. The analytic approach, using logistic regression, was appropriate for the exploratory objectives of identifying potential pathways rather than estimating causal effect sizes. Because ‘Do not want to answer’ responses were coded within indices rather than treated as a separate category, any resulting bias is likely toward the null. Sensitivity analyses confirmed that this did not meaningfully affect the direction or interpretation of results. Although a majority of participants from the second round reported campaign exposure, causal attribution of knowledge or attitude change remains uncertain. Nonetheless, the consistent directionality of associations across indices and survey rounds supports the credibility and interpretive value of the observed relationships.

## Conclusions

In this large survey of MSM in the era of U=U and treatment as prevention, we identified a nuanced interplay between cognitive understanding and emotional responses, consistent with a dual-process model of HIV-related stigma. Stronger HIV knowledge was associated with fewer negative attitudes toward people living with HIV, yet modestly greater HIV-related worry—particularly among younger respondents. Individuals with higher knowledge were also more likely to consider forming relationships with people living with HIV, while openness about sexual orientation consistently emerged as a determinant of greater knowledge, less worry, and fewer negative attitudes.

These patterns suggest plausible causal pathways linking knowledge, worry, and attitudes, though confirmation requires longitudinal investigation. Extending international evidence, our findings demonstrate that persistent misconceptions about HIV transmission remain prevalent among HIV-negative MSM in Sweden. Given the interconnectedness of LGBTQA+ and MSM communities worldwide, similar dynamics are likely in comparable high-income contexts. Even in settings where transmission risk is virtually eliminated by effective treatment, stigma persists through complex emotional and social mechanisms. Future anti-stigma strategies should therefore integrate both cognitive and affective dimensions, combining U=U education with approaches that address underlying fears and internalized prejudice.

The distinct clustering and construct validity of the indices further support the robustness of these underlying cognitive and affective domains, suggesting that the instruments developed here may be adapted for use in other contexts for comparative research on HIV stigma and related psychosocial processes.

## Supplementary Information

Below is the link to the electronic supplementary material.

Supplementary file1 (PDF 145 KB)

Supplementary file2 (PDF 171 KB)

Supplementary file3 (PDF 101 KB)

Supplementary file4 (PDF 215 KB)

## Data Availability

In commitment to transparency, anonymized study data is available upon reasonable request and consideration of participant confidentiality and data protection regulations. Parties should direct requests to the corresponding author.
