# Supplementary material for: Attitudes, Knowledge, and Worry About HIV in the U=U Era: A Campaign with Before-After Surveys Among HIV-Negative Men Who Have Sex with Men in Sweden
Source: AIDS Behav. 2026 Feb 19;30(6):1699–712. doi: 10.1007/s10461-025-04972-9 (PMC13303794; doi:10.1007/s10461-025-04972-9)
Supplement: Supplementary file 1 — Supplementary file1 (PDF 145 KB) [file 10461_2025_4972_MOESM1_ESM.pdf]

Appendix 1 – Question sets and response coding for index creation

Negative attitudes (index questions): positive index for more negative attitudes

Do you in any way describe your preference for HIV-positive or HIV-negative partners on your dating or cruising profile?

Yes, always (1)
[Coded as 1]
Yes, often (2)
[Coded as 1]
Yes, sometimes (3)
[Coded as 1]
No (4)
[Coded as 0]
Do not want to answer (5)
[Coded as 0]

Does it happen that you ask about HIV status **before** sex?

Yes, always (1)
[Coded as 0]
Before we have sex so I can cancel if the person is HIV positive (1)
[Coded as 1]
Before we have sex so I can be extra careful / just have safer sex (2)
[Coded as 0]
No (3)
[Coded as 0]
Do not want to answer (4)
[Coded as 0]

Does it happen that you ask about HIV status **before** you start a relationship?

Yes, I would not start a relationship with someone who is HIV positive (1)
[Coded as 1]
Yes, I would not start a relationship with someone who did not know their status (2)
[Coded as 0]
Yes, I can see myself having a relationship with someone who is HIV positive but would like to know (3)
[Coded as 0]
No, it doesn't matter (4)
[Coded as 0]
Do not want to answer (5)
[Coded as 0]

Do you think you could have a relationship with someone living with HIV?

Yes (1)
[Coded as 0]
Doubtful (2)
[Coded as 1]
No (3)
[Coded as 1]
Do not want to answer (4)
[Coded as 0]

How do you think about people who are open about living with HIV when seeking new partners?
(*Select all valid options*)

Good, it shows that they are honest. It makes me more interested. (1)
[Coded as 0]
Good, if you know you are living with HIV and taking medicine you cannot transmit HIV (2)
[Coded as 0]
Good that they are honest, but I'm not interested (3)
[Coded as 0]
I don't get it - it repels people (4)
[Coded as 1]
I don't understand - I wouldn't be open myself if I had HIV (5)
[Coded as 1]
Other, please specify: (6)
[Coded as 0]
No opinion (7) (Single answer)
[Coded as 0 for all]
Do not want to answer (8) (Single answer)
[Coded as 0 for all]

What do you think about people who are **not** open about living with HIV when seeking a new partner?
(*Select all valid options*)

Bad, it shows that they can't be trusted (1)
[Coded as 1]
Doesn't matter, if someone knows they are HIV positive and take medication they can't transmit HIV anyway (2)
[Coded as 0]
Okay, better to tell it when you get to know someone (3)
[Coded as 0]
I would like to report them (4)
[Coded as 1]
Not strange, I wouldn't be open about it myself if I had HIV (5)
[Coded as 0]
Other, please specify: (6)
[Coded as 0]
No opinion (7) (Single answer)
[Coded as 0]
Do not want to answer (8) (Single answer)
[Coded as 0 for all]

What do you think about the duty to inform about HIV at unmeasurable virus levels is lifted?
(*Select all valid options*)

Good, if the person can not transmit HIV (1)
[Coded as 0]
Good, because I don't need to know (2)
[Coded as 0]
Bad, I want to know regardless of whether someone is considered able to transmit HIV or not (3)
[Coded as 0]
Bad, if you know you have HIV then you should always tell your partners about it before sex (4)
[Coded as 0] (Explicitly omitted due to non-congurence with other index variables)
Bad, you can never know for sure (5)
[Coded as 0] (Explicitly omitted due to non-congurence with other index variables)
I always take PrEP, so it doesn't matter (6)
[Coded as 0]
I always use a condom / have safe(r) sex, so it doesn't matter (7)
[Coded as 0]
OK, it's my responsibility to protect myself anyway (8)
[Coded as 0]
Other, please specify (9)
[Coded as 0]
Do not want to answer (10) (Single answer)
[Coded as 0 for all]

What do you think about the obligation to use a condom at sex when living with HIV with undetectable viral levels can be removed?

Good, if the person can not transmit HIV (1)
[Coded as 0]
Good, because I don't want to know (2)
[Coded as 0]
Bad, I want to know regardless of whether someone is considered able to transmit HIV or not (3)
[Coded as 1]
Bad, if you know you have HIV then you should always tell your partner about it before sex (4)
[Coded as 1]
Bad, you can never know for sure (5)
[Coded as 0]
I always take PrEP, so it doesn't matter (6)
[Coded as 0]
I always use a condom / have safe(r) sex, so it doesn't matter (7)
[Coded as 0]
OK, it's my responsibility to protect myself anyway (8)
[Coded as 0]
Other, please specify (9) (Single answer)
[Coded as 0]
Do not want to answer (10)
[Coded as 0 for all]

Which of the following statements do you agree with?
(*Select all valid options*)

Men who are HIV negative do <u>not</u> understand what it means to have HIV. (1)
[Coded as 0]
Even among men who have sex with men, there is discrimination against those with HIV. (2)
[Coded as 0]
Most men who are HIV negative are afraid to spend time with someone who has HIV. (3)
[Coded as 1]
I would only have sex with someone whose HIV status is the same as mine (4)
[Coded as 0]
I would expect an <u>HIV positive man to tell</u> me he was HIV positive before we had sex (5)
[Coded as 0]
I would expect an <u>HIV negative man to tell</u> me he was HIV negative before we had sex (6)
[Coded as 0]
None of the above statements (7)
[Coded as 0 for all]

Knowledge (index questions): positive index for more knowledge

Do you know if the risk of someone transmitting HIV is **reduced <u>or</u> stopped** by…?
(*Select all valid options*)

Being on drug treatment for HIV? (1)
[Coded as 1]
Having non-measurable virus load? (2)
[Coded as 1]
Not sharing syringes? (3)
[Coded as 1]
Using a condom? (4)
[Coded as 1]
Removing the foreskin of the penis? (5)
[Coded as 1]
Not being the recipient of semen? (6)
[Coded as 1]
By taking PrEP? (7)
[Coded as 1]
By taking PEP? (8)
[Coded as 1]
Only having oral sex? (9)
[Coded as 1]
Do not want to answer (10) (Single answer)
[Coded as 0 for all]

Did you know that the duty to inform about HIV can be removed at undetectable virus levels?

Yes (1)
[Coded as 1]
No (2)
[Coded as 0]
Do not want to answer (3)
[Coded as 0]

Did you know that the obligation to use a condom at sex when living with HIV with undetectable virus levels can be removed?

Yes (1)
[Coded as 1]
No (2)
[Coded as 0]
Do not want to answer (3)
[Coded as 0]

Have you heard of **<u>PrEP</u>**?
<u>Pre-</u>* *exposure prophylaxis *is medication that is taken* ***<u>before sex</u>*** *to reduce the risk of HIV transmission when having sex with someone you think can transmit HIV*

Yes (1)
[Coded as 1]
No (2)
[Coded as 0]
Do not want to answer (3)
[Coded as 0]

Have you heard of **<u>PEP</u>**?
<u>Post-</u>* *exposure prophylaxis *is medication that you take* ***<u>after sex</u>*** *to reduce the risk of HIV transmission when you have had sex with someone who you believe can transmit HIV*

Yes (1)
[Coded as 1]
No (2)
[Coded as 0]
Do not want to answer (3)
[Coded as 0 for all]

Worry 2021 (index questions): positive index for more worry

What aspects are you most worried about when it comes to being HIV positive?
(*Select all valid options*)

I would have to take medication (1)
[Coded as 1]
What others would think about me (12)
[Coded as 1]
How others would treat me (14)
[Coded as 1]
It would be difficult if my family found out (2)
[Coded as 1]
It would be difficult if my friends found out (3)
[Coded as 1]
It would be difficult to have tell my sex partners (4)
[Coded as 1]
I could spread HIV to others (5)
[Coded as 1]
My sex life would be affected (6)
[Coded as 1]
I think it will be harder to find a partner (19)
[Coded as 1]
It could complicate my role in relation to my partner(s) (20)
[Coded as 1]
It would negatively affect my self-confidence (21)
[Coded as 1]
I'm afraid it would affect my body / appearance (22)
[Coded as 1]
I could get sick (7)
[Coded as 1]
I would be afraid to become alone (8)
[Coded as 1]
I'd be scared to die (9)
[Coded as 1]
It is a condition that never disappears (10)
[Coded as 1]
Other, please specify: (11)
[Coded as 0]
I am not worried (15) (Single answer)
[Coded as 0 for all]
Do not know / Do not want to answer (23) (Single answer)
[Coded as 0 for all]

Are you worried about being or becoming HIV positive?
(*Choose a number from 0-10; where 0 is not at all worried and 10 is very worried*)

0-3
[Coded as 0]
4-10
[Coded as 1]

Worry 2020 (alternative options, also includes scale):

What aspects are you most worried about when it comes to being HIV positive?
(*Select all valid options*)

I would have to take medication (1)
[Coded as 1]
It would be difficult if my family found out (2)
[Coded as 1]
It would be difficult if my friends found out (3)
[Coded as 1]
It would be difficult to have tell my sex partners (4)
[Coded as 1]
I could spread HIV to others (5)
[Coded as 1]
My sex life would be affected (6)
[Coded as 1]
I could get sick (7)
[Coded as 1]
I would be afraid to become alone (8)
[Coded as 1]
I'd be scared to die (9)
[Coded as 1]
It is a condition that never disappears (10)
[Coded as 1]
Other, please specify: (11)
[Coded as 0]

Are you worried about being or becoming HIV positive?
(*Choose a number from 0-10; where 0 is not at all worried and 10 is very worried*)

0-4
[Coded as 0]
5-10
[Coded as 1]
