# Supplementary material for: Attitudes, Knowledge, and Worry About HIV in the U=U Era: A Campaign with Before-After Surveys Among HIV-Negative Men Who Have Sex with Men in Sweden
Source: AIDS Behav. 2026 Feb 19;30(6):1699–712. doi: 10.1007/s10461-025-04972-9 (PMC13303794; doi:10.1007/s10461-025-04972-9)
Supplement: Supplementary file 2 — Supplementary file2 (PDF 171 KB) [file 10461_2025_4972_MOESM2_ESM.pdf]

Appendix 2 – Bivariate Distributions by Outcome Domain

This appendix presents detailed bivariate comparisons of respondent characteristics by each of the four main study outcomes: HIV-related negative attitudes (Table A2.1), HIV knowledge (Table A2.2), HIV-related worry (Table A2.3), and willingness to form a relationship with a person living with HIV (Table A2.4). Percentages are column percentages within each outcome category. P-values are derived from Pearson's chi-squared tests.

Table A2.1. Distribution of sociodemographic and behavioral characteristics by level of HIV-related negative attitudes (Less vs. More)

Characteristic	*Less n (%)*	*More n (%)*	*p-value*
Knowledge index (quintiles)			<0.001
Lowest	185 (12%)	394 (25%)	
Lower	246 (16%)	403 (26%)	
Middle	307 (20%)	321 (21%)	
Higher	369 (24%)	250 (16%)	
Highest	442 (29%)	183 (12%)	
Worry index (quintiles)			<0.001
Lowest	336 (22%)	254 (16%)	
Lower	362 (23%)	276 (18%)	
Middle	345 (22%)	282 (18%)	
Higher	293 (19%)	330 (21%)	
Highest	213 (14%)	409 (26%)	
Country of birth (Sweden/Other)	1,303 (85%) / 228 (15%)	1,320 (87%) / 199 (13%)	0.154
University education (Any/None)	1,000 (65%) / 538 (35%)	844 (55%) / 696 (45%)	<0.001
Relationship status (Steady/Open/None)	475 (31%) / 334 (22%) / 740 (48%)	522 (34%) / 286 (18%) / 743 (48%)	0.051
Uses dating or cruising apps	1,288 (84%)	1,357 (88%)	0.004
≥6 sexual partners (past year)	497 (32%)	328 (21%)	<0.001
Age ≥65 years	188 (13%)	271 (19%)	<0.001
Open about sexual identity	1,125 (73%)	739 (48%)	<0.001
Acknowledges HIV-stigma among MSM	1,104 (71%)	823 (53%)	<0.001
Ever had sex with HIV-positive partner	545 (35%)	190 (12%)	<0.001

Note: Percentages are column percentages within each outcome category. P-values from Pearson's chi-squared tests.

Table A2.2. Distribution of sociodemographic and behavioral characteristics by level of HIV knowledge (Less vs. More)

Characteristic	*Less n (%)*	*More n (%)*	*p-value*
Negative attitude index (quintiles)			<0.001
Lowest	246 (16%)	647 (42%)	
Lower	329 (21%)	327 (21%)	
Middle	309 (20%)	209 (13%)	
Higher	314 (20%)	190 (12%)	
Highest	346 (22%)	183 (12%)	
Worry index (quintiles)			<0.001
Lowest	328 (21%)	262 (17%)	
Lower	378 (24%)	260 (17%)	
Middle	300 (19%)	327 (21%)	
Higher	259 (17%)	364 (23%)	
Highest	279 (18%)	343 (22%)	
Country of birth (Sweden/Other)	1,327 (88%) / 188 (12%)	1,296 (84%) / 239 (16%)	0.012
University education (Any/None)	1,138 (73%) / 412 (27%)	706 (46%) / 822 (54%)	<0.001
Relationship status (Steady/Open/None)	429 (28%) / 370 (24%) / 757 (49%)	568 (37%) / 250 (16%) / 726 (47%)	<0.001
Uses dating or cruising apps	1,337 (86%)	1,308 (86%)	0.570
≥6 sexual partners (past year)	530 (34%)	295 (19%)	<0.001
Age ≥65 years	156 (11%)	303 (21%)	<0.001
Open about sexual identity	1,132 (73%)	732 (47%)	<0.001
Acknowledges HIV-stigma among MSM	1,222 (79%)	705 (46%)	<0.001
Ever had sex with HIV-positive partner	559 (36%)	176 (11%)	<0.001

Note: Percentages are column percentages within each outcome category. P-values from Pearson's chi-squared tests.

Table A2.3. Distribution of sociodemographic and behavioral characteristics by level of HIV-related worry (Less vs. More)

Characteristic	*Less n (%)*	*More n (%)*	*p-value*
Knowledge index (quintiles)			<0.001
Lowest	370 (24%)	209 (13%)	
Lower	344 (22%)	305 (20%)	
Middle	291 (19%)	337 (22%)	
Higher	286 (19%)	333 (21%)	
Highest	253 (16%)	372 (24%)	
Negative attitude index (quintiles)			<0.001

Lowest	512 (33%)	381 (24%)	
Lower	358 (23%)	298 (19%)	
Middle	267 (17%)	251 (16%)	
Higher	216 (14%)	288 (19%)	
Highest	191 (12%)	338 (22%)	
Country of birth (Sweden/Other)	1,339 (88%) / 184 (12%)	1,284 (84%) / 243 (16%)	0.002
University education (Any/None)	1,010 (65%) / 537 (35%)	834 (54%) / 697 (46%)	<0.001
Relationship status (Steady/Open/None)	450 (29%) / 310 (20%) / 796 (51%)	547 (35%) / 310 (20%) / 687 (44%)	<0.001
Uses dating or cruising apps	1,382 (89%)	1,263 (83%)	<0.001
≥6 sexual partners (past year)	473 (30%)	352 (23%)	<0.001
Age ≥65 years	164 (11%)	295 (20%)	<0.001
Open about sexual identity	926 (60%)	938 (61%)	0.481
Acknowledges HIV-stigma among MSM	1,105 (71%)	822 (53%)	<0.001
Ever had sex with HIV-positive partner	350 (22%)	385 (25%)	0.110

Note: Percentages are column percentages within each outcome category. P-values from Pearson's chi-squared tests.

Table A2.4. Distribution of sociodemographic and behavioral characteristics by willingness to form a relationship with a person living with HIV (No/Doubtful vs. Yes)

Characteristic	*No/Doubtful n (%)*	*Yes n (%)*	*p-value*
Negative attitude index (quintiles)			<0.001
Lowest	180 (12%)	595 (37%)	
Lower	322 (22%)	445 (28%)	
Middle	261 (18%)	234 (15%)	
Higher	344 (23%)	196 (12%)	
Highest	380 (26%)	143 (9%)	
Knowledge index (quintiles)			<0.001
Lowest	449 (30%)	130 (8%)	
Lower	393 (26%)	256 (16%)	
Middle	283 (19%)	345 (21%)	
Higher	211 (14%)	408 (25%)	
Highest	151 (10%)	474 (29%)	
Worry index (quintiles)			<0.001
Lowest	251 (17%)	339 (21%)	
Lower	287 (19%)	351 (22%)	
Middle	274 (18%)	353 (22%)	
Higher	292 (20%)	331 (21%)	
Highest	383 (26%)	239 (15%)	
Country of birth (Sweden/Other)	1,267 (87%) / 182 (13%)	1,356 (85%) / 245 (15%)	0.029
University education (Any/None)	1,069 (66%) / 540 (34%)	775 (53%) / 694 (47%)	<0.001
Relationship status (Steady/Open/None)	494 (31%) / 357 (22%) / 762 (47%)	503 (34%) / 263 (18%) / 721 (48%)	0.006
Uses dating or cruising apps	1,353 (84%)	1,292 (88%)	0.010
≥6 sexual partners (past year)	532 (33%)	293 (20%)	<0.001
Age ≥65 years	172 (11%)	287 (21%)	<0.001
Open about sexual identity	1,256 (78%)	608 (41%)	<0.001
Acknowledges HIV-stigma among MSM	1,225 (76%)	702 (47%)	<0.001
Ever had sex with HIV-positive partner	614 (38%)	121 (8%)	<0.001

Note: Percentages are column percentages within each outcome category. P-values from Pearson's chi-squared tests.
