# Supplementary material for: Attitudes, Knowledge, and Worry About HIV in the U=U Era: A Campaign with Before-After Surveys Among HIV-Negative Men Who Have Sex with Men in Sweden
Source: AIDS Behav. 2026 Feb 19;30(6):1699–712. doi: 10.1007/s10461-025-04972-9 (PMC13303794; doi:10.1007/s10461-025-04972-9)
Supplement: Supplementary file 3 — Supplementary file3 (PDF 101 KB) [file 10461_2025_4972_MOESM3_ESM.pdf]

Appendix 3 – Descriptive distribution of survey responses by item (total pooled sample N = 3,100)

**Attitudes and behaviors**		
**Describes preference for HIV-positive or HIV-negative partners on dating or cruising profiles**	**Count**	**Percent**
Yes, always	126	4.06
Yes, often	65	2.10
Yes, sometimes	121	3.90
No	2667	86.03
Do not want to answer	121	3.90
Total	3100	100.00
**Asking about HIV status before sex**	**Count**	**Percent**
Before we have sex so I can cancel if the person is HIV positive	584	18.84
Before we have sex so I can be extra careful / just have safer sex	786	25.35
No	1600	51.61
Do not want to answer	130	4.19
Total	3100	100.00
**Asks about HIV status before starting a relationship**	**Count**	**Percent**
Yes, I would not start a relationship with someone who is HIV positive	807	26.03
Yes, I would not start a relationship with someone who did not know their status	429	13.84
Yes, I can see myself having a relationship with someone who is HIV positive but would like to know	858	27.68
No, it doesn't matter	663	21.39
Do not want to answer	343	11.06
Total	3100	100.00
**Would consider relationship with someone living with HIV**	**Count**	**Percent**
Yes	1613	52.03
Doubtful	847	27.32
No	535	17.26
Do not want to answer	105	3.39
Total	3100	100.00

**Opinion of people who are open about living with HIV when seeking new partners? (Multiple response)**	**Count**	**Percent**
Good, it shows that they are honest. It makes me more interested.	1061	34.65
Good, if you know you are living with HIV and taking medicine you cannot transmit HIV	1351	44.12
Good that they are honest, but I'm not interested	1318	43.04
I don't get it - it repels people	22	0.72
I don't understand - I wouldn't be open myself if I had HIV	34	1.11
No opinion (Single response)	157	5.13
Do not want to answer (Single response)	22	0.72

**Opinion of people who are not open about living with HIV when seeking a new partner? (Multiple response)**	**Count**	**Percent**
Bad, it shows that they can't be trusted	1688	56.95
Doesn't matter, if someone knows they are HIV positive and take medication they can't transmit HIV anyway	574	19.37
Okay, better to tell it when you get to know someone	768	25.91
I would like to report them [to the authorities]	252	8.50
Not strange, I wouldn't be open about it myself if I had HIV	197	6.65
No opinion (Single response)	185	6.24
Do not want to answer (Single response)	44	1.48

**Opinion on waived duty to inform about HIV at unmeasurable viral load (Multiple response)**	**Count**	**Percent**
Good, if the person cannot transmit	1547	53.64
Good, because I don't need to know	376	13.04
Bad, I want to know regardless of whether someone is considered able to transmit HIV or not	711	24.65
Bad, if you know you have HIV then you should always tell your partners about it before sex	539	18.69
Bad, you can never know for sure	351	12.17
I always take PrEP, so it doesn't matter	84	2.91
I always use a condom / have safe(r) sex, so it doesn't matter	238	8.25
OK, it's my responsibility to protect myself anyway	642	22.26

**Opinion on waived obligation to use a condom at sex when living with HIV with undetectable viral load (Multiple response)**	**Count**	**Percent**
Good, if the person can not transmit HIV	346	40.80
Good, because I don't want to know	51	6.01
Bad, I want to know regardless of whether someone is considered able to transmit HIV or not	188	22.17
Bad, if you know you have HIV then you should always tell your partner about it before sex	110	12.97
Bad, you can never know for sure	106	12.50
I always take PrEP, so it doesn't matter	25	2.95
I always use a condom / have safe(r) sex, so it doesn't matter	90	10.61
OK, it's my responsibility to protect myself anyway	142	16.75
Do not want to answer (Single response)	29	3.42

**Agreement with statements about HIV stigma** ***(Select all valid options)***	**Count**	**Percent**
Men who are HIV negative do not understand what it means to have HIV	1451	47.08
Even among men who have sex with men, there is discrimination against those with HIV.	1927	62.52
Most men who are HIV negative are afraid to spend time with someone who has HIV.	1062	34.46
I would only have sex with someone whose HIV status is the same as mine	628	20.38

I would expect an HIV positive man to tell me he was HIV positive before we had sex	2057	66.74
I would expect an HIV negative man to tell me he was HIV negative before we had sex	715	23.20
None of the above statements (Single response)	230	7.46

**Ever used PrEP – n=3085**	**Count**	**Percent**
Yes	227	7.36
No	2856	92.58
Do not want to answer	2	0.06
**Ever used PEP – n=3083**	**Count**	**Percent**
Yes	63	2.04
No	3018	97.89
Do not want to answer	2	0.06

**Knowledge**		
**Knowledge of reduced transmission risk of HIV (Multiple response)**	**Count**	**Percent**
Being on drug treatment for HIV?	1648	53.16
Having non-measurable virus load?	1776	57.29
Not sharing syringes?	1768	57.03
Using a condom?	2661	85.84
Not being the recipient of semen?	988	31.87
By taking PrEP?	1444	46.58
By taking PEP?	456	14.71
Only having oral sex?	605	19.52
Do not want to answer (Single response)	195	6.29

**Knows that the duty to inform about HIV can be waived when living with HIV with undetectable viral loads**	**Count**	**Percent**
Yes	1270	40.97
No	1760	56.77
Do not want to answer	70	2.26
Total	3100	100.00
**Knows that the obligation to use a condom during sex can be waived when living with HIV with undetectable viral loads**	**Count**	**Percent**
Yes	770	24.84
No	2249	72.55
Do not want to answer	81	2.61
Total	3100	100.00
**Have you heard of PrEP ?**	**Count**	**Percent**
Yes	2122	68.45
No	963	31.06
Do not want to answer	15	0.48
Total	3100	100.00
**Knows of PEP**	**Count**	**Percent**
Yes	1212	39.10
No	1871	60.35
Do not want to answer	17	0.55
Total	3100	100.00
**Recognizes any U=U messaging**	**Count**	**Percent**
Yes	854	27.55
No	2246	72.45
Total	3100	
**Encountered terms / campaign? (Multiple response)**	**Count**	**Percent**
U = U (Utan virus = Utan risk)	178	4.83
U = U (Undetectable = Untransmittable)	371	10.07
O = O (Omätbart = Oöverförbart)	263	7.14
'*Smittfri hiv*'	626	16.99
No (Single answer)	2246	60.97
**2020 only: Which term do you think best communicates to others about the effects of HIV treatment? (Single answer)**	**Count**	**Percent**
U = U (Utan virus = Utan risk)	321	14.30
U = U (Undetectable = Untransmittable)	278	12.39
O = O (Omätbart = Oöverförbart)	335	14.93
'*Smittfri hiv*'	644	28.70
Other	67	2.99
Do not want to answer	599	26.69
Total	2244	
**2021 only: Encountered study-campaign**	**Count**	**Percent**
Yes	610	53.89
No	522	46.11
Total	1132	

**Worry**		
**2021: Most worrying aspects about being HIV positive (Multiple response)**	**Count**	**Percent**
I would have to take medication	477	42.14
What others would think about me	422	37.28
How others would treat me	414	36.57

It would be difficult if my family found out	400	35.34
It would be difficult if my friends found out	304	26.86
It would be difficult to have tell my sex partners	399	35.25
I could spread HIV to others	502	44.35
My sex life would be affected	515	45.49
I think it will be harder to find a partner	480	42.40
It could complicate my role in relation to my partner(s)	293	25.88
It would negatively affect my self-confidence	375	33.13
I'm afraid it would affect my body or appearance	185	16.34
I could get sick	512	45.23
I would be afraid to become alone	307	27.12
I'd be scared to die	281	24.82
It is a condition that never disappears	448	39.58
I am not worried (Single response)	39	3.45
Do not know / Do not want to answer (Single response)	30	2.65

**2020: Most worrying aspects about being HIV positive (Multiple response)**	**Count**	**Percent**
I would have to take medication	894	49.12
It would be difficult if my family found out	837	45.99
It would be difficult if my friends found out	672	36.92
It would be difficult to have tell my sex partners	792	43.52
I could spread HIV to others	998	54.84
My sex life would be affected	993	54.56
I could get sick	1050	57.69
I would be afraid to become alone	719	39.51
I'd be scared to die	499	27.42
It is a condition that never disappears	996	54.73

**Worry about being or becoming HIV positive? *(Likert scale 0-10)***	**Obs**	**Mean**	**Std. Dev.**	**Min**	**Max**
	3100	3.75	3.05	0	10
