# Supplementary material for: Attitudes, Knowledge, and Worry About HIV in the U=U Era: A Campaign with Before-After Surveys Among HIV-Negative Men Who Have Sex with Men in Sweden
Source: AIDS Behav. 2026 Feb 19;30(6):1699–712. doi: 10.1007/s10461-025-04972-9 (PMC13303794; doi:10.1007/s10461-025-04972-9)
Supplement: Supplementary file 4 — Supplementary file4 (PDF 215 KB) [file 10461_2025_4972_MOESM4_ESM.pdf]

Appendix 4. Multivariable logistic regression plots for 2021 subsample including campaign exposure

This appendix presents coefficient plots from the 2021 multivariable logistic regression models assessing associations between exposure to the anti-HIV stigma campaign and the four outcome domains: HIV-related negative attitudes, knowledge, worry, and relationship willingness. n = 1,099

Factors associated with more negative attitudes toward HIV, adjusted for campaign exposure (reference: less negative attitudes)

Variable	Value	n	aOR (95% CI)	p–value
**Worry Index Score**		1099	1.27 (1.20, 1.35)	<0.001
**Knowledge Index Score**		1099	0.83 (0.75, 0.91)	<0.001
**Country of birth**	Other	131	Reference	
	Sweden	968	0.93 (0.60, 1.42)	0.731
**University education**	No	441	Reference	
	Yes	658	1.03 (0.76, 1.39)	0.864
**Relationship status**	Steady partner	277	Reference	
	Open relationship	366	0.89 (0.59, 1.33)	0.561
	No partner	456	1.00 (0.68, 1.48)	0.987
**Uses dating/cruising apps**	Non–user	140	Reference	
	User	959	1.32 (0.85, 2.06)	0.225
**Sexual partners (past year)**	None	152	Reference	
	1–2	376	0.97 (0.61, 1.53)	0.881
	3–5	275	0.96 (0.59, 1.55)	0.867
	6+	296	0.66 (0.41, 1.07)	0.095
**Age categories**	18–24	53	Reference	
	25–34	143	0.84 (0.41, 1.69)	0.625
	35–44	198	0.93 (0.46, 1.84)	0.836
	45–54	236	0.97 (0.48, 1.93)	0.926
	55–64	270	1.08 (0.54, 2.14)	0.823
	65+	199	1.70 (0.82, 3.50)	0.150
**City**	Stockholm	434	Reference	
	Major city	164	0.91 (0.60, 1.36)	0.640
	Medium–sized city	172	1.55 (1.02, 2.37)	0.042
	Smaller city	171	1.57 (1.03, 2.39)	0.036
	Small town/country	158	1.27 (0.82, 1.96)	0.280
**Acknowledges HIV–stigma among MSM**	No	408	Reference	
	Yes	691	0.83 (0.61, 1.13)	0.226
**Ever had sex with HIV–positive partner**	No	790	Reference	
	Yes	309	0.52 (0.37, 0.73)	<0.001
**Openness about sexuality**	Homosexual & Open	565	Reference	
	Homosexual & Not Open	123	1.15 (0.74, 1.80)	0.531
	Bi/pansexual & Open	86	2.03 (1.21, 3.43)	0.008
	Bi/pansexual & Not Open	285	2.11 (1.46, 3.06)	<0.001
	Other & Open	20	0.68 (0.23, 1.89)	0.470
	Other & Not Open	20	1.95 (0.70, 5.87)	0.214
**Campaign Exposure**	No	507	Reference	
	Yes	592	0.75 (0.56, 1.00)	0.046

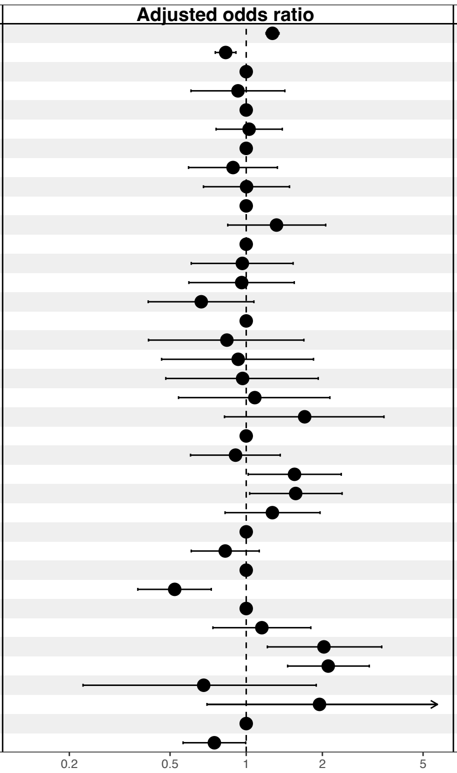

Factors associated with more knowledge about HIV, adjusted for campaign exposure (reference: less knowledge)

Variable	Value	n	aOR (95% CI)	p–value
**Worry Index Score**		1099	1.10 (1.03, 1.16)	0.002
**Negative Attitudes Index Score**		1099	0.82 (0.74, 0.90)	<0.001
**Country of birth**	Other	131	Reference	
	Sweden	968	1.57 (1.01, 2.44)	0.044
**University education**	No	441	Reference	
	Yes	658	2.10 (1.57, 2.81)	<0.001
**Relationship status**	Steady partner	277	Reference	
	Open relationship	366	1.33 (0.88, 1.99)	0.176
	No partner	456	1.44 (0.97, 2.15)	0.071
**Uses dating/cruising apps**	Non–user	140	Reference	
	User	959	1.09 (0.70, 1.70)	0.710
**Sexual partners (past year)**	None	152	Reference	
	1–2	376	1.17 (0.73, 1.87)	0.523
	3–5	275	1.15 (0.71, 1.87)	0.567
	6+	296	1.32 (0.80, 2.15)	0.276
**Age categories**	18–24	53	Reference	
	25–34	143	1.08 (0.52, 2.23)	0.838
	35–44	198	1.02 (0.50, 2.05)	0.966
	45–54	236	0.72 (0.35, 1.45)	0.360
	55–64	270	0.89 (0.44, 1.78)	0.745
	65+	199	0.86 (0.41, 1.78)	0.679
**City**	Stockholm	434	Reference	
	Major city	164	0.73 (0.48, 1.12)	0.147
	Medium–sized city	172	0.95 (0.62, 1.46)	0.832
	Smaller city	171	0.71 (0.46, 1.08)	0.112
	Small town/country	158	0.71 (0.46, 1.09)	0.118
**Acknowledges HIV–stigma among MSM**	No	408	Reference	
	Yes	691	1.96 (1.46, 2.65)	<0.001
**Ever had sex with HIV–positive partner**	No	790	Reference	
	Yes	309	1.92 (1.35, 2.75)	<0.001
**Openness about sexuality**	Homosexual & Open	565	Reference	
	Homosexual & Not Open	123	0.64 (0.41, 1.02)	0.058
	Bi/pansexual & Open	86	0.83 (0.49, 1.43)	0.506
	Bi/pansexual & Not Open	285	0.41 (0.29, 0.60)	<0.001
	Other & Open	20	0.36 (0.13, 1.00)	0.044
	Other & Not Open	20	0.68 (0.25, 1.88)	0.457
**Campaign Exposure**	No	507	Reference	
	Yes	592	1.40 (1.05, 1.86)	0.021

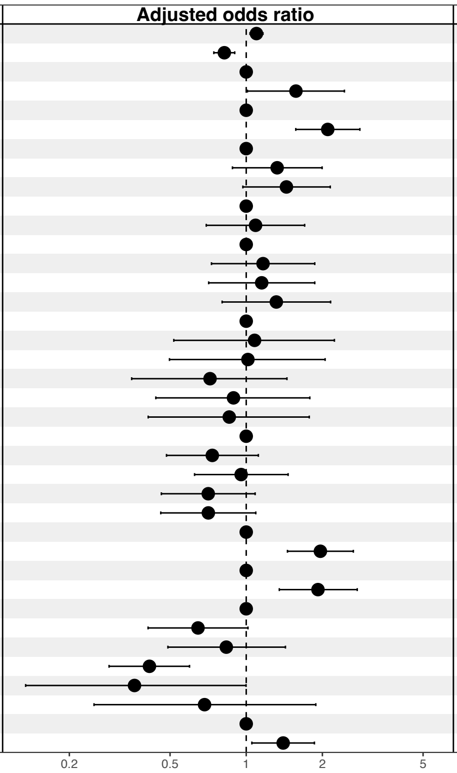

Appendix 4. Multivariable logistic regression plots for 2021 subsample including campaign exposure

Factors associated with more worry about HIV, adjusted for campaign exposure (reference: less worry)

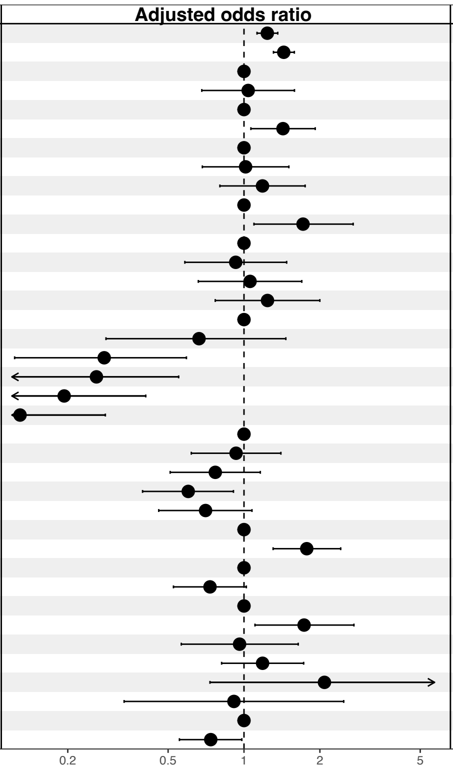

Variable	Value	n	aOR (95% CI)	p–value
**Knowledge Index Score**		1099	1.24 (1.13, 1.36)	<0.001
**Negative Attitudes Index Score**		1099	1.44 (1.31, 1.58)	<0.001
**Country of birth**	Other	131	Reference	
	Sweden	968	1.04 (0.68, 1.59)	0.859
**University education**	No	441	Reference	
	Yes	658	1.43 (1.06, 1.92)	0.017
**Relationship status**	Steady partner	277	Reference	
	Open relationship	366	1.01 (0.68, 1.51)	0.942
	No partner	456	1.18 (0.80, 1.75)	0.395
**Uses dating/cruising apps**	Non–user	140	Reference	
	User	959	1.71 (1.10, 2.71)	0.019
**Sexual partners (past year)**	None	152	Reference	
	1–2	376	0.93 (0.58, 1.48)	0.749
	3–5	275	1.06 (0.66, 1.70)	0.818
	6+	296	1.24 (0.77, 2.00)	0.378
**Age categories**	18–24	53	Reference	
	25–34	143	0.66 (0.28, 1.47)	0.324
	35–44	198	0.28 (0.12, 0.59)	0.001
	45–54	236	0.26 (0.11, 0.55)	<0.001
	55–64	270	0.19 (0.09, 0.41)	<0.001
	65+	199	0.13 (0.06, 0.28)	<0.001
**City**	Stockholm	434	Reference	
	Major city	164	0.93 (0.62, 1.40)	0.728
	Medium–sized city	172	0.77 (0.51, 1.16)	0.212
	Smaller city	171	0.60 (0.40, 0.91)	0.016
	Small town/country	158	0.70 (0.46, 1.08)	0.106
**Acknowledges HIV–stigma among MSM**	No	408	Reference	
	Yes	691	1.77 (1.30, 2.42)	<0.001
**Ever had sex with HIV–positive partner**	No	790	Reference	
	Yes	309	0.73 (0.53, 1.02)	0.067
**Openness about sexuality**	Homosexual & Open	565	Reference	
	Homosexual & Not Open	123	1.73 (1.11, 2.73)	0.017
	Bi/pansexual & Open	86	0.96 (0.56, 1.64)	0.882
	Bi/pansexual & Not Open	285	1.18 (0.82, 1.72)	0.375
	Other & Open	20	2.09 (0.73, 6.90)	0.191
	Other & Not Open	20	0.91 (0.33, 2.49)	0.856
**Campaign Exposure**	No	507	Reference	
	Yes	592	0.74 (0.55, 0.98)	0.037

Factors associated with willingness to form a relationship with a person living with HIV, adjusted for campaign exposure (reference: no/doubtful willingness)

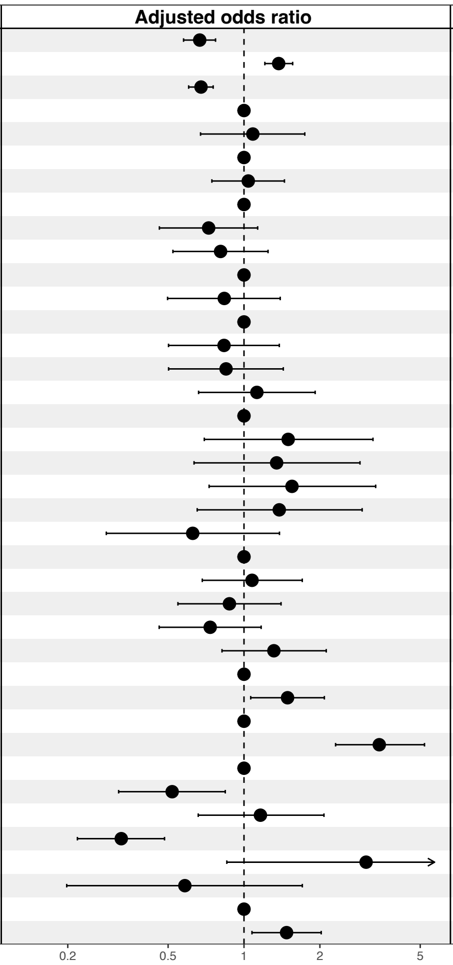

Variable	Value	n	aOR (95% CI)	p–value
**Worry Index Quintile**		1099	0.67 (0.58, 0.77)	<0.001
**Knowledge Index Quintile**		1099	1.37 (1.21, 1.56)	<0.001
**Attitudes Index Quintile**		1099	0.68 (0.60, 0.75)	<0.001
**Country of birth**	Other	131	Reference	
	Sweden	968	1.08 (0.67, 1.74)	0.740
**University education**	No	441	Reference	
	Yes	658	1.04 (0.75, 1.45)	0.817
**Relationship status**	Steady partner	277	Reference	
	Open relationship	366	0.72 (0.46, 1.13)	0.158
	No partner	456	0.81 (0.52, 1.25)	0.334
**Uses dating/cruising apps**	Non–user	140	Reference	
	User	959	0.84 (0.50, 1.39)	0.492
**Sexual partners (past year)**	None	152	Reference	
	1–2	376	0.83 (0.50, 1.38)	0.480
	3–5	275	0.85 (0.50, 1.43)	0.540
	6+	296	1.12 (0.66, 1.92)	0.665
**Age categories**	18–24	53	Reference	
	25–34	143	1.50 (0.70, 3.25)	0.303
	35–44	198	1.35 (0.63, 2.88)	0.440
	45–54	236	1.55 (0.73, 3.33)	0.258
	55–64	270	1.38 (0.65, 2.94)	0.401
	65+	199	0.63 (0.28, 1.38)	0.245
**City**	Stockholm	434	Reference	
	Major city	164	1.08 (0.68, 1.70)	0.752
	Medium–sized city	172	0.88 (0.55, 1.40)	0.579
	Smaller city	171	0.73 (0.46, 1.17)	0.193
	Small town/country	158	1.31 (0.82, 2.12)	0.261
**Acknowledges HIV–stigma among MSM**	No	408	Reference	
	Yes	691	1.49 (1.06, 2.08)	0.020
**Ever had sex with HIV–positive partner**	No	790	Reference	
	Yes	309	3.44 (2.31, 5.20)	<0.001
**Openness about sexuality**	Homosexual & Open	565	Reference	
	Homosexual & Not Open	123	0.52 (0.32, 0.84)	0.008
	Bi/pansexual & Open	86	1.16 (0.66, 2.08)	0.608
	Bi/pansexual & Not Open	285	0.33 (0.22, 0.48)	<0.001
	Other & Open	20	3.05 (0.85, 13.01)	0.103
	Other & Not Open	20	0.58 (0.20, 1.70)	0.320
**Campaign Exposure**	No	507	Reference	
	Yes	592	1.48 (1.08, 2.02)	0.016
